# Substitutive Press-Bolster and Press-Ram Models for the Virtual Estimation of Stamping-Tool Cambering

**DOI:** 10.3390/ma15010279

**Published:** 2021-12-30

**Authors:** Farshad Abbasi, Alex Sarasua, Javier Trinidad, Nagore Otegi, Eneko Saenz de Argandoña, Lander Galdos

**Affiliations:** 1 Advanced Material Processes Research Group, Department of Mechanical and Industrial Production, Mondragon University, Loramendi 4, 20500 Mondragon, Spain; jtrinidad@mondragon.edu (J.T.); notegi@mondragon.edu (N.O.); esaenzdeargan@mondragon.edu (E.S.d.A.); lgaldos@mondragon.edu (L.G.); 2 Matrici S. Coop., Ugaldeguren II, Parcela 14-V, 48170 Zamudio, Spain; asarasua@matrici.com

**Keywords:** tool cambering, press-deflection-measuring system, substitute model, sheet-metal forming (SMF), try-out and production

## Abstract

Today’s stamping simulations are realized by ignoring the elastic deformation of the press and tooling system through the assumption of a rigid behavior and a perfect press stroke. However, in reality, the press and tool components deform elastically and are one of the major error sources for the final adjustment and blue-spotting of the dies. In order to tackle this issue, a new approach is proposed in this study that substitutes the press stiffness by means of a substitutive model composed of cost-effective shell and beam elements. The substitute model was calibrated using full-scale measurements, in which a 20,000 kN trial press was experimentally characterized by measuring its deformation under static loads. To examine the robustness of the substitute model, a medium-size tool and a large-size tool were simulated together with the substitutive model. To this end, a B-pillar tool was re-machined based on the substitute-model results and a new cambering procedure was proposed and validated throughout the blue-painting procedure. The newly developed substitute model was able to replicate the global stiffness of the press with a high accuracy and affordable calculation time. The implementation of the findings can aid toolmakers in eliminating most of the reworking and home-line trials.

## 1. Introduction

Never before has the car industry been as challenging, interesting, and demanding as it is today. New and advanced techniques are being introduced with increased speed in order to achieve sustainable development, improved performance, and heightened customer satisfaction. This has led to increasing competition in an almost ever-expanding car market. In recent years, significant attention has been paid to the elastic deformation of the press-tool system and its effect on the forming simulations, mainly because is an important factor in the estimation of tool cambering during the die-tool trial phases. Due to significant improvements in material models, today’s stamping-process simulations are realized by ignoring the elastic deformation of the press and tooling system by assuming a rigid behavior with a perfect press stroke, in order to reduce the numerical cost as well as the ramp-out time during the tool’s trial phase. This system, including the bolster, ram, and drawing cushion, and its tools deform elastically during the stamping process, thereby forming a clearance gap between the die and the punch, which leads to a non-homogeneous pressure distribution on the part. These elastic deformations are responsible for much of the disparity between reality and the simulations, as well as for the problems that arise when dies are mounted on production presses with different elastic characteristics, a fact which is not considered in the process FEM simulation. This is becoming a critical aspect for automotive OEMs and TIER1s, since the early-stage trial in the tool shop is performed by a trial-and-error procedure leading to an additional final adjustment prior to their use in the production presses.

**Figure 1:**
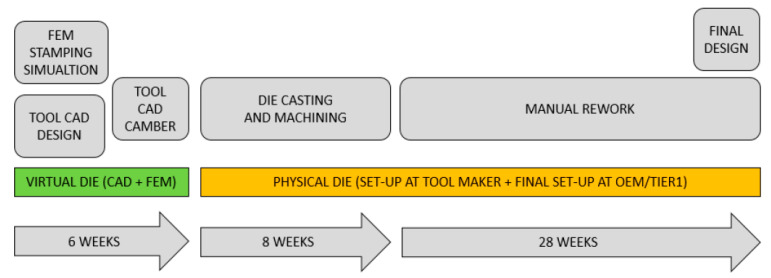
Traditional approach for design and manufacturing of dies (given times are for a skin panel although they vary with each component) [1].

The development and manufacture of stamping dies is one of the costliest and most time-consuming tasks of new car projects. A simplified description of the process that consists of a virtual phase followed by a physical phase is shown in Figure 1. The virtual phase entails CAD- and FE-stamping simulations and tool cambering followed by manual rework in order to counteract the elastic deformations that occur in the real process.

Once the virtual phase is completed, the die is casted, milled and manually adjusted for a robust production process, first in the tool maker and then in its production press at the final stage. The rework is part of a trial process in the tool shop that is often followed by subsequent rework steps in the production press. Rework of the forming surfaces is performed either by adding or removing material from the forming surfaces of the stamping die. The process is highly manual and time consuming, and thus costly. It can require up to 30 weeks for a single die, and hundreds of dies are manufactured worldwide for each car project. Some research has found that approximately 350–500 h of manual work are needed to finish a stamping die so that the required results are obtained [2]. During this time, an initial rough polishing is performed in order to adjust the draw-in according to the simulation and to obtain a sound part. For this purpose, draw beads and shims are reworked after the polishing step. Then, die spotting is performed, which is the most expensive and time-consuming phase of the trial. In particular, the die spotting is empirical and almost entirely dependent on the toolmaker’s experience, and it ends in a time-consuming manual rework process until good parts are produced. Within this step, the tools’ active surfaces are manually modified until a blue-painted sheet is stamped and all active tool surfaces appear in blue. After this step, the stamped components are measured using CMM and digitalization systems, and the tool-compensation steps starts. These compensations are mainly for spring-back corrections and elastic-deformation cambering, since tools deform during formation and their active surfaces need to be cambered in the opposite direction in order to obtain a part with the required tolerances. After several loops of correction and die spotting, the tool is approved by the TIER1 or OEM and, if needed, coatings are applied.

To compensate for the elastic behavior of a specific stamping press, it is necessary to identify the press-specific characteristics. The most important ones are the applied forces, the deflection behavior of the moving bolster, ram and drawing cushion, as well as the tilt rigidity of the ram and cushion. To this end, different measurement systems [2,3,4,5,6,7] have been developed over the last few years based on VDI 3193 [8], VDI 3194 [9] and DIN 55189 [10] standards. Volkswagen, in cooperation with Fraunhofer IWU, has developed a multi-functional press-measuring system called the press-fingerprint-tool to retrieve the most important press characteristics within only a few hours of measuring time [11]. A total of 51 tactile-displacement sensors were attached to the measuring frames on the upper and lower tool in order to measure the deflection of the ram and bolster. Moreover, Behrens et al. developed a new method for measuring press deflections, both under static and dynamic loading [12]. The same group presented a method for measuring press deflections using static bars by mounting a real tool in the press [13]. Additionally, it is also worth noting that there is no standard for measuring the press and tool stiffness at the same time.

One of the main limitations when including the behavior of the press is the complexity of modeling the real geometry of the entire press, which in some cases is not available in the workshop. Furthermore, the tool meshes have to be extremely fine in order to preserve the contact-surface smoothness, so the size of the problem is increased by an order of magnitude. In this context, the numerical cost becomes a major factor for toolmakers, particularly when dealing with a large-size press and tools. The goal is to minimize the time and effort in trials by using model-reduction techniques and developing substitute models for the press during the tool-planning stage. Pilthammar et al. developed a bolster-substitute model using topology optimization in which the measurements of the bolster deformations were carried out with force transducers and an ARAMIS 3D optical-measurement system [14]. The press table was then inversely modeled by topology optimization using the recorded results as the boundary conditions. Finally, the press table was coupled with an FE-model of a die in order to demonstrate its influence on the deformations. More recently, Zgoll et al. developed substitutive press models that enabled the fast simulation of the stamping-die deflection using an uncoupled approach [15,16]. The mounting surfaces of the bolster and ram were supported by groups of linear-elastic springs, whose free ends were fixed. The stiffness values of the springs were calibrated during parameter optimization, for which the measured deflection was used as the target. However, further examination of different tools is necessary to evaluate the robustness of the aforementioned substitute models.

As detailed above, advancements beyond the state-of-the-art press-deflection-measuring systems and substitute models for tool cambering are incremental. However, there is hardly any work devoted to the development of a simple, fast and affordable substitute model that is able to replicate the stiffness given by both the press bolster and press ram, simultaneously. To address this scientific gap, the major aim of this research was to develop a cost-effective methodology able to reproduce the stiffness of the whole press by means of a substitutive model composed of low-cost shell and beam elements that corresponds to the real dimensions of the press. The newly developed substitute model was calibrated against full-scale measurements, in which a 20,000 kN trial press was experimentally characterized by measuring its elastic deformation under static loads. To evaluate the robustness of the newly developed substitute model, a medium-size tool and a large-size tool were simulated together with the substitutive press model and the deformations in the active surfaces of both tools were compared with the full-model-simulation results. Finally, a new cambering procedure was proposed in order to reduce the setup time and cost by shortening the trial phase of a stamping die. To this end, a B-pillar car tool was re-machined based on the results obtained from the substitute-model simulation and the new cambering procedure was validated through the blue-painting procedure. 

## 2. Experimental Setup for Press Measurement

**Figure 2:**
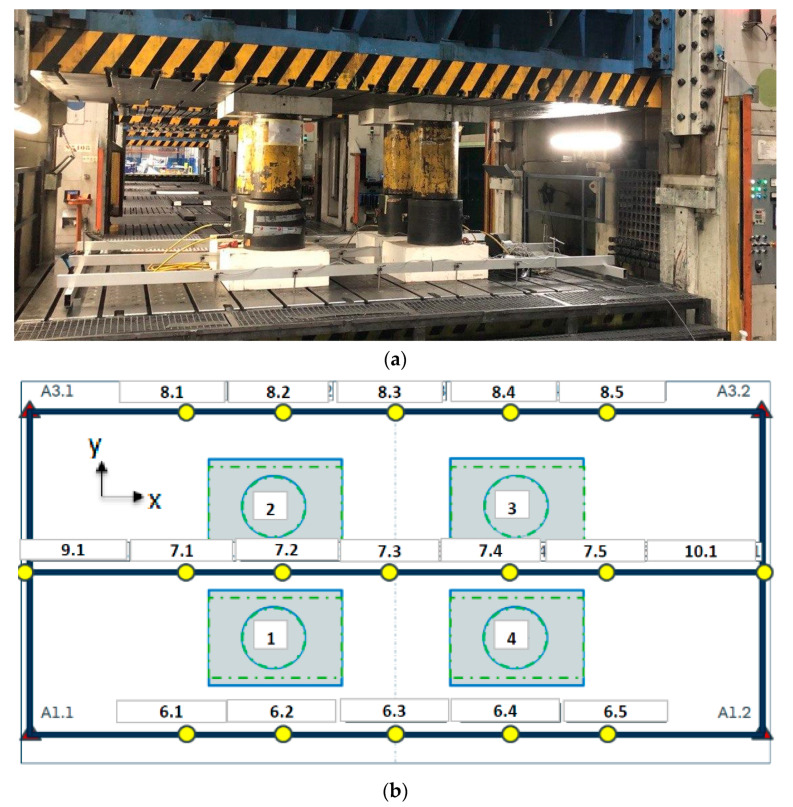
(**a**) A general view of the trial press and setup for measuring system of the table and (**b**) Position of pillars and dial gauges on measuring frame.

Press measurements were performed on a single-action mechanical trial press of 20,000 kN, with bolster dimensions of 5000 × 2750 mm. The press was held in its Bottom Dead Centre (BDC) where it was fully closed against four steel pillars that were placed on the press table in a rectangular arrangement in the middle of the bolster. Each pillar had a diameter of 420 mm and a length of 700 mm. The pillars were progressively loaded by four hydraulic cylinders as shown in Figure 2a. Hardened steel plates of dimensions 800 mm × 600 mm × 290 mm were placed between the pillars and the press mounting surfaces to prevent possible damage to the press. The applied force was controlled by adjusting the filling pressure of the hydraulic cylinders. A total force of 16,000 kN was used for the press measurement in this study. Three pre-run tests were performed in order to stabilize the static measurements of the displacements. The measuring frame consisted of three measuring bars, one in the middle, one in the front and one on the back side of the press table as shown in Figure 2a. The same procedure was used for the ram by attaching the measuring rams to the ram surface. A total of 17 dial gauges (HBM-WA20 series LVDT sensors) attached to the measuring frame were used to measure the displacement of the table and ram. The sensors had a measuring range of 0–20 mm and a linearity deviation of ±0.2% between the start and end points (including hysteresis by reference to nominal sensitivity). The uncertainty study that was performed using these sensors at the laboratory scale and at a stroke of 10 mm (mid stroke) showed a repeatability of approximately ±0.005 mm and a standard deviation of 0.0012. It should be noted that the measuring bars shown in Figure 2a are 300 mm shorter than the table at both left and right sides and the bars are shifted 350 mm at front and back sides. Moreover, there are four points visible in Figure 2b without dial gauges attached, which are the supports of the measuring frame, making them the reference points for all of the other measured values. Lastly, software was used to collect the displacement data from the dial gauges.

## 3. Full Press and Tool Numerical Simulation

**Figure 3:**
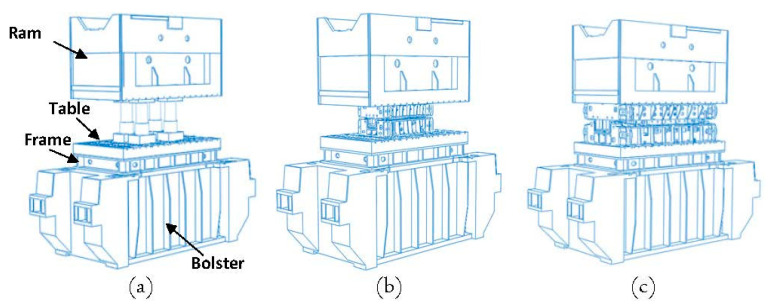
Full-model closed-tool assembly of (**a**) four pillars, (**b**) B-pillar and (**c**) Lateral tools.

In order to obtain the press and tool deflections and to quantify the tool cambering, a fully closed tool-state FE simulation was set up in ABAQUS. In the first step, a full model of the trial press, including four steel pillars of the measuring system, was developed as shown in Figure 3a. The steel pillars were positioned on the FE model just as they were in the experiment. The geometries of the FE model were the same as the experimental assembly illustrated in Figure 2a. However, because the global stiffness of the press was dominated mainly by the bolster and ram, the guiding columns of the press were neglected in the FE models.

**Figure 4:**
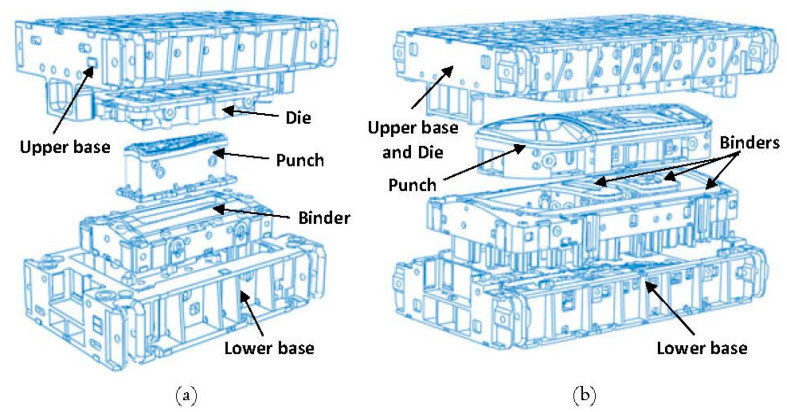
Exploded view of (**a**) B-pillar and (**b**) Lateral tools.

In order to validate the FE model, the press bolster and ram displacement results were compared to the measured values that were obtained in Section 2. In the next step, a full closed-assembly model of a medium-size tool (B-pillar reinforcement) and a large-size tool (side panel, lateral) mounted on the trial press were generated in ABAQUS as shown in Figure 3b,c. The displacements of the press (bolster and ram) and tools (die, punch and binders) that were obtained from the FE models will be used later to validate the newly developed substitute model. For the sake of abbreviation, the B-pillar and Lateral car panels are designated by “B-pillar” and “Lateral”, respectively. The exploded views of the B-pillar and Lateral tools are shown in Figure 4. Dimensions of the lower and upper base were 2460 mm × 1500 mm and 4480 mm × 2380 mm for the B-pillar and Lateral tools, respectively.

In relation to the boundary condition, the MPC constraint was applied to the four support regions of the bolster. However, the bolster was allowed to rotate with respect to the x- and y-axes. The four corners of the ram were restricted from moving in the z-axis to imitate the behavior of the guiding columns of the press. The maximum force of 16,000 kN, 12,000 kN and 20,000 kN was applied linearly on the connecting regions of the ram for the four-pillars case, the B-pillar and Lateral tools, respectively. To guide the binders in relation to the die, all of the wear plates in the binders were restricted from moving in z-axis.

The bed cushion force was transmitted from the displacement cylinders via a pressure pad and pressure pins to the blank holder (binder) of the drawing die. The cushion force was set to be 3000 kN and 5000 kN for the B-pillar and Lateral tools, respectively. In addition, a surface-to-surface contact algorithm followed by a small sliding property was assigned to all of the contacting surfaces in the model. The penalty method with the friction constant of 0.1 was included in the contact pairs. To avoid possible numerical divergence due to the complex contact condition between the die and the punch in B-pillar and Lateral tools, mesh refinement was conducted at the tool-contact interfaces. However, relatively coarse mesh was used for the regions away from the contact interface using a biasing approach. A total number of 1,350,000, 1,720,000 and 2,352,000 linear tetrahedral solid elements of type C3D4 were generated for the four-pillars case, the B-pillar and Lateral tools, respectively. The press bolster and ram were assigned with typical structural steel while the tooling components, e.g., the punch, die and binders, were assigned with cast iron GG70L. The mechanical properties are presented in Table 1.

## 4. Proposed Substitute Model and Calibration Procedure

The main objective of the present study was to develop a simple, fast and universally applicable substitute model using model-reduction techniques and low-cost FE elements such as shell and beam elements. The substitute model must be able to reproduce the elastic deflection of the press and predict the cambering of the active tool surfaces as accurately as possible. In addition, heavy duty trial presses are subjected to continuously changing process conditions. During their lifetime, different dies and tools of all sizes are mounted on them. In this regard, the substitute model must be unique in its properties and must replicate the stiffness given by the press, regardless of the size of the tooling.

**Figure 5:**
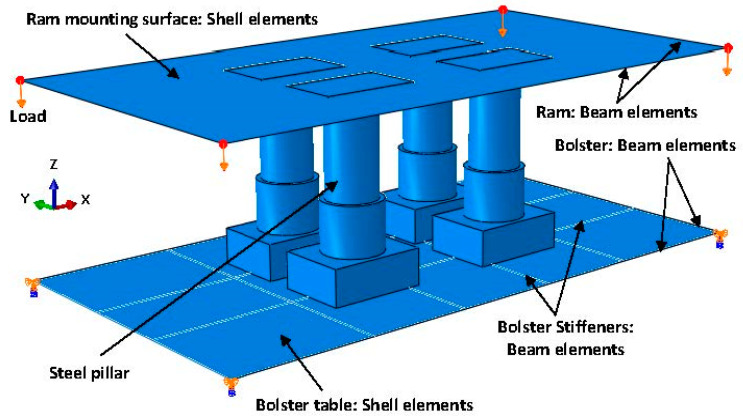
Press-bolster- and ram-substitute model.

**Figure 6:**
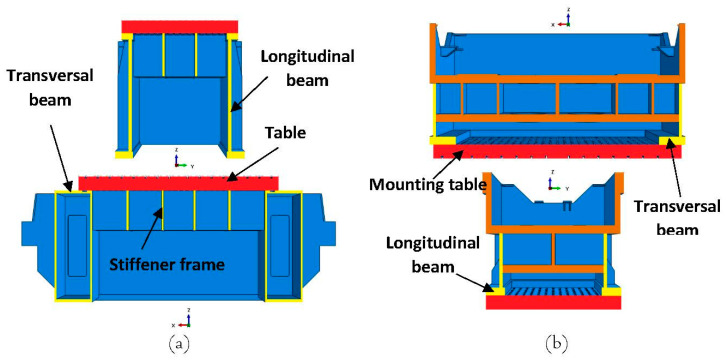
CAD-model characterization of (**a**) press bolster and (**b**) press ram.

The newly developed substitute model consists of a press bolster and a ram and is shown in Figure 5. The steel pillars were positioned on the FE-substitute model just as they were in the experiment in order to validate the displacement predicted by the substitute model. As shown in Figure 5, the model was composed of beam and shell elements that replicated the stiffness given by the press. In order to identify the main structure of the press and introduce the beam and shell properties to the substitute model, the full CAD model of the press was characterized along the x- and y-axes as shown in Figure 6. As seen in the figure, the main structure of the press bolster was made of two longitudinal C-profile beams and two transversal rectangular box-profile beams that were connected together with a frame stiffener, which the table was mounted on. The cross section of these beams (marked with yellow color in Figure 6 was generated and assigned to the beam elements of the bolster in the substitute model. However, the press ram possessed a more complicated structure than the bolster, as seen in Figure 6b. In addition to the main L-profile beams joined together along the x- and y-axes, there were several ribs and thick plates inside the ram (marked with brown color in Figure 6b that were connected to the main beams in order to ensure maximum rigidity and minimize the deflection of the entire press construction. The effective stiffnesses of these plates were superimposed with the stiffnesses of the longitudinal and transversal beam elements in the substitute model during a calibration procedure in which the measured displacement was used as a target.

In addition, both the bolster table and the mounting surface of the ram (marked with red color in Figure 6 were modeled with elastic shell elements with thicknesses corresponding to the real dimensions of the press, which were 350 mm and 280 mm for the bolster and ram, respectively.

As for the boundary conditions, the bolster was supported at the four corner nodes that were restricted from moving in all directions and were free to rotate around the x- and y-axes, similar to the full-model simulations described in Section 3. The finite sliding surface-to-surface contact was applied to all of the contacting surfaces in the model. However, a tie contact was implemented on the contacting regions of beam and shell elements, which allowed for rapid convergence of the model and took the initial thickness and offset of the shell elements underlying the surface into account. The load was applied linearly to the four edges of the ram, because the substitute-model boundaries were the mounting surfaces of the press bolster and ram, as shown in Figure 5. 

However, in some cases the technical drawings and real geometry of the entire press are not available in the workshop. In this context, the main advantage of the newly proposed substitutive model in this study is that the real 3D geometry of the press is not necessary, and the calibration procedure is very straightforward. It should be noted that the displacement of the substitute model is mainly dominated by the stiffness of the longitudinal and stiffener beams and the thickness of the shell elements. Therefore, the main parameters in the calibration methodology are the moment of inertia of the longitudinal and stiffener beams and the thickness of the shell elements, which can be easily modified in the FE-substitute model until a best fit with the measured displacement is achieved by using a suitable calibration procedure (e.g., Optimization tools available in MATLAB linked to ABAQUS software). In this way, specific press-substitute models can be generated for any type of measured press, as proposed in this study.

## 5. Results and Discussion

### 5.1. Bolster/Ram Experimental Elastic Deflection

**Figure 7:**
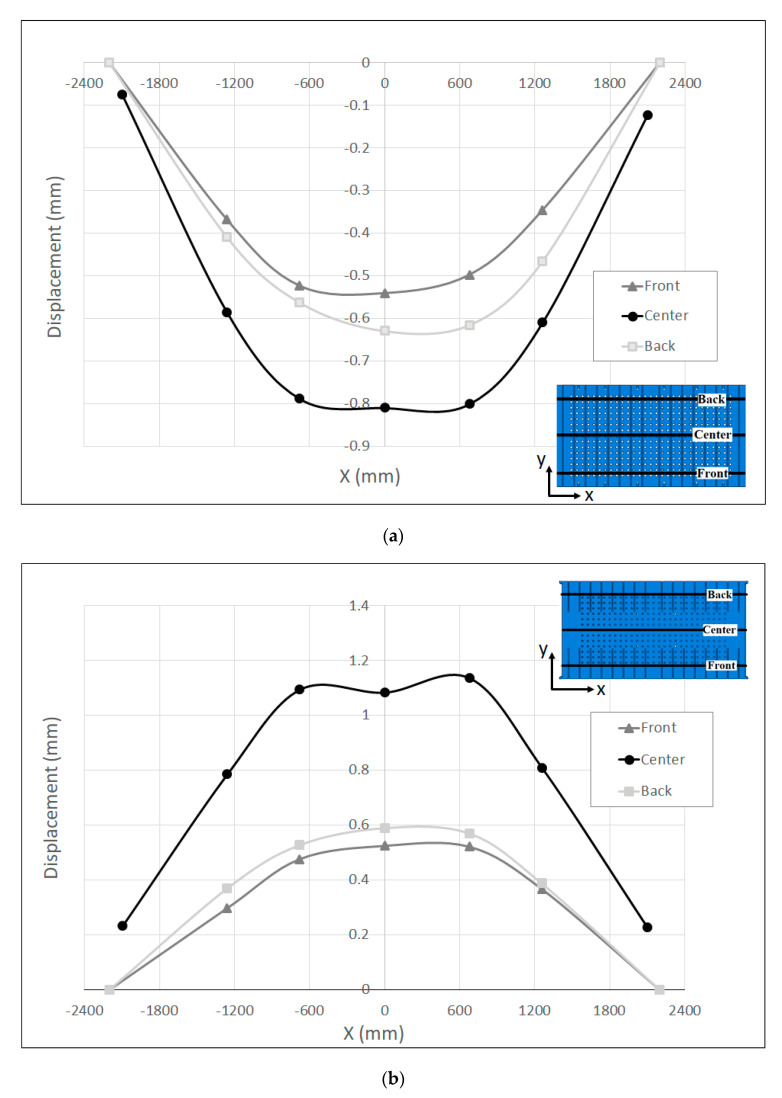
Measured displacement along the measuring bars at the (**a**) Table and (**b**) Ram.

The resulting measurement of the displacement along the measuring bars that were attached to the front, center and back sides of the press bolster and ram are shown in Figure 7a,b, respectively. Along the center lines, the maximum displacement was about 0.81 mm in the bolster and 1.1 mm in the ram, relative to the reference points of the measuring bars (see Figure 2b). These results demonstrate the lesser rigidity of the ram compared to the bolster due to the different structural properties of the press bolster and ram. As seen in Figure 6, the table was mounted on a stiffener frame inside the press bolster, while the mounting surface of the ram was only supported by the longitudinal and transversal beams without any reinforcement in the middle in order to restrict its deformation. This may be a reason why the ram shows a greater displacement compared to the bolster. However, the asymmetrical behavior of the displacement was seen along the front and back measuring bars for both the bolster and ram. Along the front lines, the maximum displacement was 0.54 mm for the bolster and 0.52 mm for the ram, while the maximum displacement amounted to 0.63 mm and 0.59 mm along the back lines for the bolster and ram, respectively. This asymmetry in displacement can be due the variation in the pre-charging of the main pillars of the press throughout the press stroke.

### 5.2. Validation of Full Press Numerical Simulation

**Figure 8:**
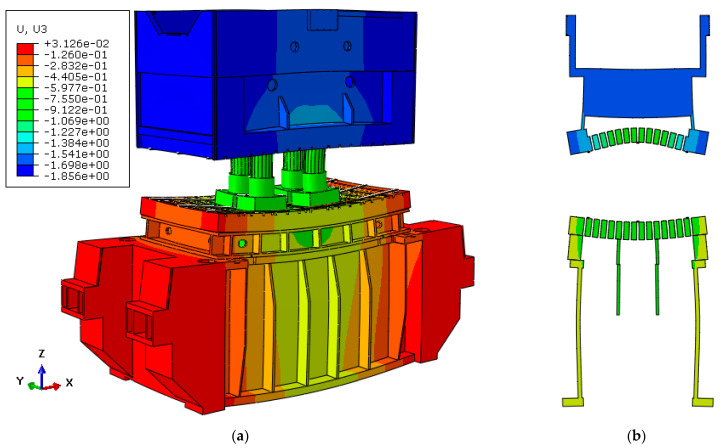
Deformation of the full model with scale factor of 300 (**a**) general view and (**b**) cut view along x-axis.

Figure 8a represents the deformation behavior of the full model under the maximum load of 16,000 kN with a scale factor of 500 obtained from ABAQUS. The cut view of the model at the middle of the press along the x-axis is shown in Figure 8b. As seen in the figures, the press table and ram were deformed in opposite direction with respect to each other while the values of the deformation were on the millimeter scale. These results show the significance of the press elasticity on the industrial SMF process and real production tools.

**Figure 9:**
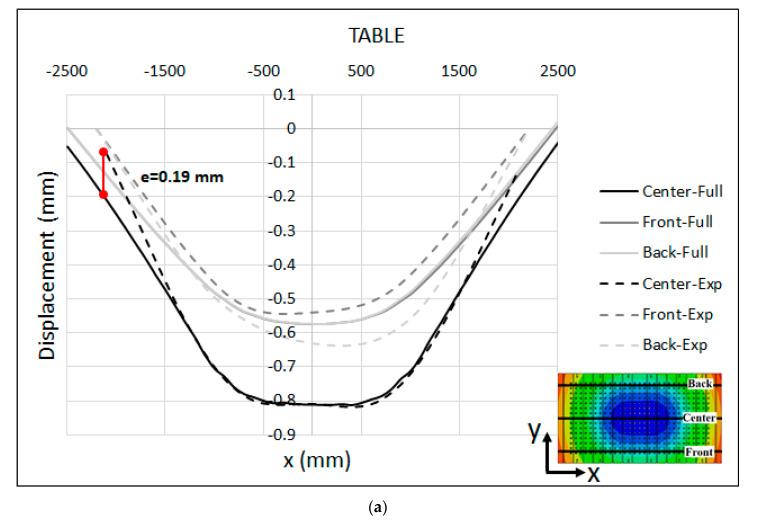

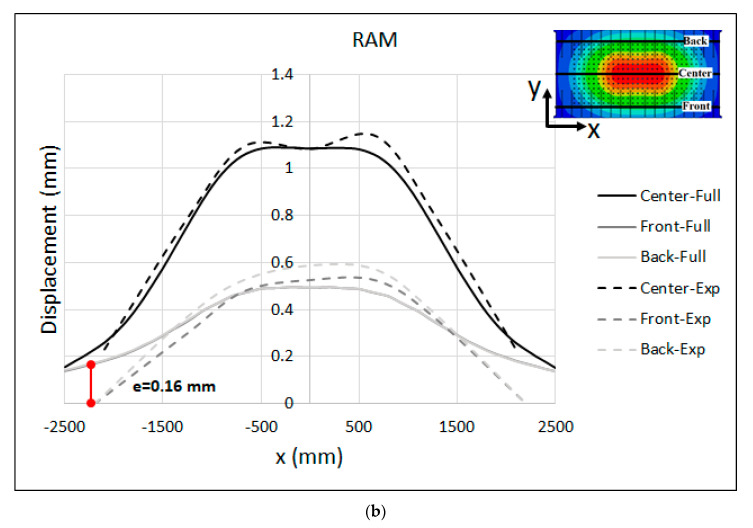
Measured displacement vs. full-model numerical simulation (adjusted) at the (**a**) Table and (**b**) Ram.

The comparison between the measured displacement and the full-model numerical simulation is shown in Figure 9a,b for the press table and ram, respectively. Because the relative movement of the surfaces and shapes adopted by the ram and table in comparison to the experimental measurements is the important aspect for tool cambering, the predicted numerical displacements were shifted so that the displacement at the middle of the table and the ram were equal to the experimental data. This adjustment enables an accurate comparison of the final resulting shapes of the surfaces. Furthermore, the displacements were plotted along three lines in the front, center and back sides of the table and ram, corresponding to the location of the measuring bars during the experimental measurement. It should be noted that the numerical results are plotted over a range of −2500 mm < X < 2500 mm. However, the measuring bars are shorter than the table, hence the measured values are displayed over a range of −2200 mm < X < 2200 mm in Figure 9.

The displacements that were predicted by the numerical simulation were in a satisfactory agreement with the measured values, confirming the validity of the FE model and the numerical-simulation result. As seen in the figure, the maximum deviation between the experimental and numerical results was about e = 0.19 mm at the left edge of the table along the centerline (X = −2200 mm), and e = 0.16 mm at the left corner of the ram (X = −2200 mm). One possible explanation for this slight deviation can be attributed to the fact that in the full-model simulation, the press pillars were not modeled. Therefore, the global rotational stiffness of these elements was not included in the model, which could explain the excessive rotation of the numerical results at the left and right sides of the press. This may also explain the error in the ram, as the opening of the guiding system of the ram due to the bending of the main press pillars was not included, which introduced more stiffness to the full numerical model than in reality.

### 5.3. Validation of the Substitute Model 

**Figure 10:**
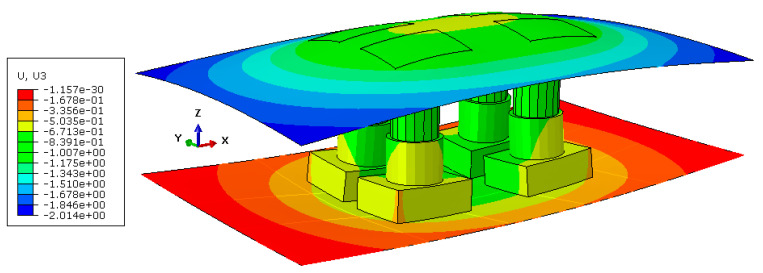
Deformed substitute model with a scale factor of 300.

**Figure 11:**
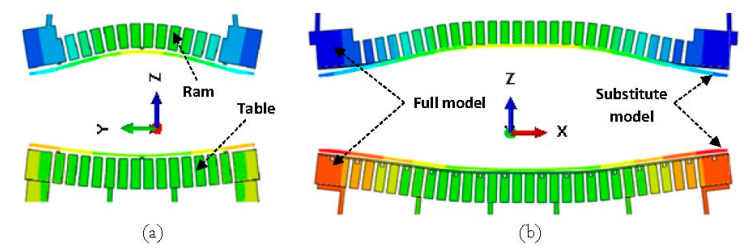
Comparison between the substitute-model- and full-model-simulation-predicted shape at the middle section of the models along the (**a**) x-axis and (**b**) y-axis.

The deformed substitute model with a scale factor of 300 and maximum applied load of 16,000 kN is shown in Figure 10. As seen in the figure, the displacements were on the millimeter scale and comparable to the results of the full model in Figure 8. In order to examine the capability of the substitute model to predict the final shape of the press, a comparison between the substitute model and the full-model simulation would be useful. To this end, a cut view of the middle section of both models along the x- and y-axes were overlaid as shown in Figure 11. As seen in the figure, the deformed shape predicted by the substitute model was in very good agreement with the full-model simulation for both the press table and ram. 

**Figure 12:**
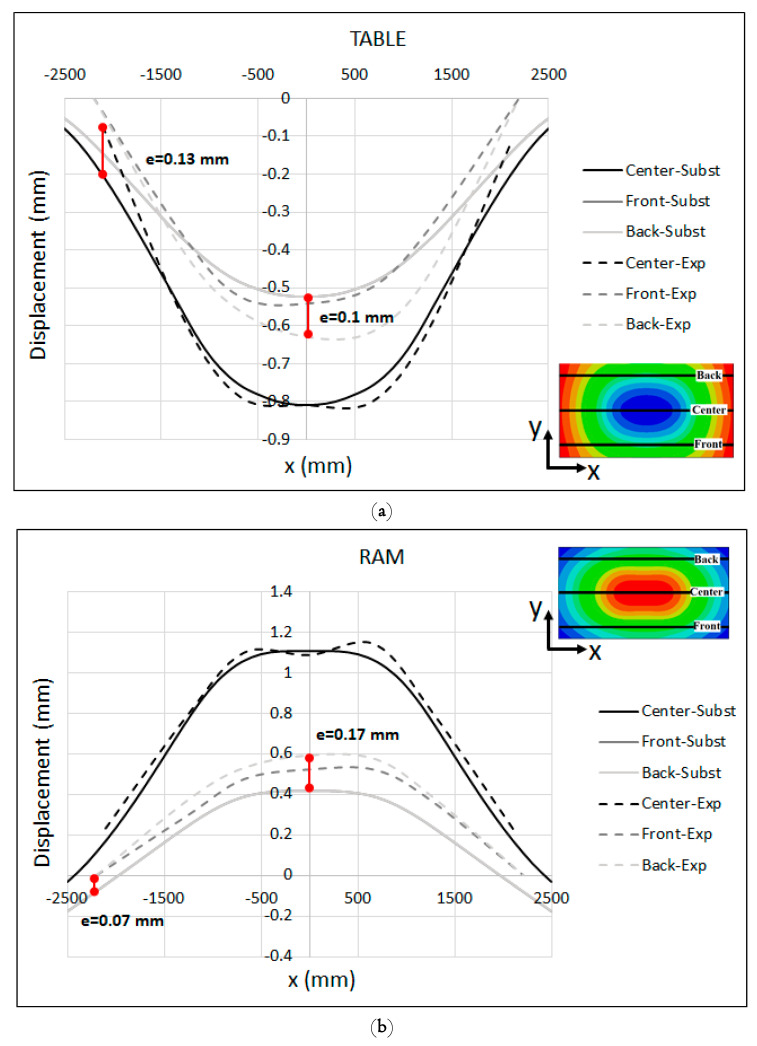
Measured displacement vs. substitute-model prediction (adjusted) at the (**a**) Table and (**b**) Ram.

To gain more insight into the results, the comparison between the measured displacements and the substitute-model predictions of the table and ram are presented in Figure 12a,b, respectively. As seen in the figures, the global shape of deformed table and ram were captured satisfactorily by the substitute model. The final shape of the table and ram is a key factor in developing a cambering strategy, since in real productions the global deformation of tooling parts (basically the die and punch) is followed by the copying of the global shape of the press table and ram. Relative to the estimated displacement, the results predicted by the substitute model were in quite good agreement with the experimental observations. For the table, the results show a maximum deviation of e = 0.13 mm between the experimental measurement and the substitute-model results at the left edge of the centerline (X = −2200 mm), and e = 0.1 mm at the midpoint of the back line (X = 0). For the ram, the maximum deviation between the experimental and substitute-model results was about e = 0.17 mm at the midpoint of the back/front lines. Specially, along the centerline of the ram, both the measured values and the substitute-model predictions were almost identical. The peak values along the centerlines of the measured results in Figure 12 are associated with the regions underneath the press bolster and mounting surface of the ram that are located between the ribs structure, where the stiffness is not enough to restrict the elastic deformations. The aforementioned ribs were not modeled in the substitute model for the sake of simplicity.

One possible reason for the slight deviation between the measured values and the substitute-model prediction can be explained by the measuring-system setup. As shown in Figure 2b, the measuring frame was supported at four points in the corners where no dial gauges were attached. These points were the reference points of the measuring system and all of the measured values were relative to these points. Hence, the displacement of these reference points was set to be zero. Another possible explanation can be attributed to the difference between the boundary condition applied to the support regions of the press-bolster-substitute model and what was happening in reality. In the substitute model, the bolster table was connected to the beam structures whose corners were restricted from moving in all directions, while in reality the bolster bases at the four regions were mounted on rigid foundations attached to the ground. This may have affected the deformation of the table, especially at corners where a tie contact was defined between the shell and beam elements constraining the table in order to track the beam-element deformation. Furthermore, as explained in Section 5.2 the guiding columns of the press were not included in the FE models for the sake of simplicity. In the real press, these columns may rotate and bend under loading and affect the global deformation of the press bolster and ram. To the best of author’s belief, further studies are needed to investigate the effect of press columns on global stiffness and the deformation of the entire press bolster and ram. The introduction of rotational stiffness to the supports of the substitutive model could be a simple way to include the effect of the real press columns.

## 6. Application of Substitutive Models Using Industrial Tools

### 6.1. Virtual Trial of Medium-Size (B-Pillar) and Large-Size (Lateral) Tools

**Figure 13:**
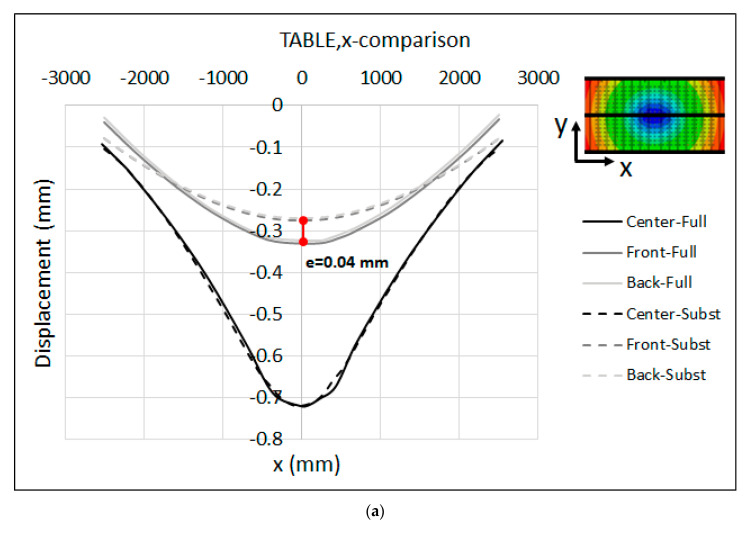

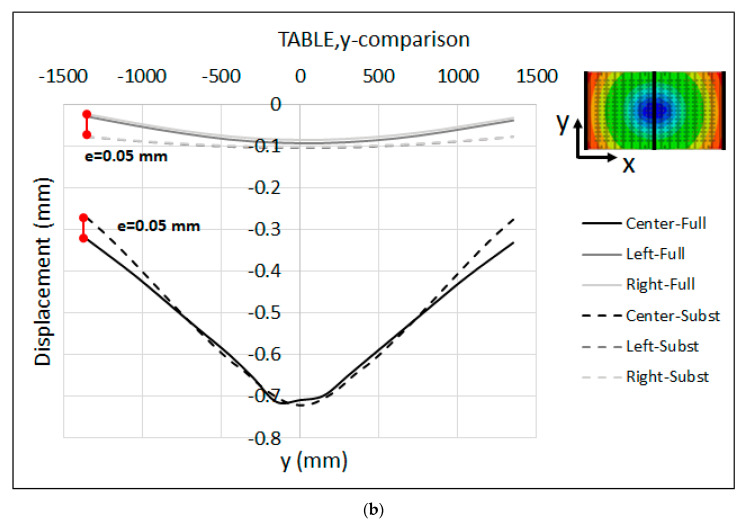
Substitute model vs. full-model displacement (adjusted) of the press TABLE along the (**a**) x-axis and (**b**) y-axis for the B-Pillar tool.

To evaluate the robustness of the substitute model against real industrial tools, a B-pillar tool (see Figure 4a), which is a medium-size tool, was modeled together with the substitute model, and the deformations in the press table, ram and tooling were compared with the full-model-simulation results. It is important to highlight the fact that the substitute model of the press with the calibrated parameters that were previously determined in Section 4 was used. Figure 13 shows the comparison between the displacements predicted by the substitute-model and full-model simulations for the press table in the x- and y-axes. Note that the displacement is plotted across the center, front and back lines along the x-axis, and the center, left and right lines along the y-axis. Moreover, the displacements predicted by the substitute model were adjusted so that the displacement at the middle of the table and ram were equal to the full-model results.

As seen in the figure, the maximum displacement at the center of the table was about 0.71 mm. Moreover, the displacements predicted by the substitute model were in a quite good agreement with the full-model-simulation results. It is interesting to note that the deformed shape of both the table and ram along the centerline was identical for both models. However, there was a maximum deviation of e = 0.04 mm at the middle point of the front/back line along the x-axis. Moreover, the maximum deviation was about e = 0.05 mm at the edge of the center, left and right lines along the y-axis.

**Figure 14:**
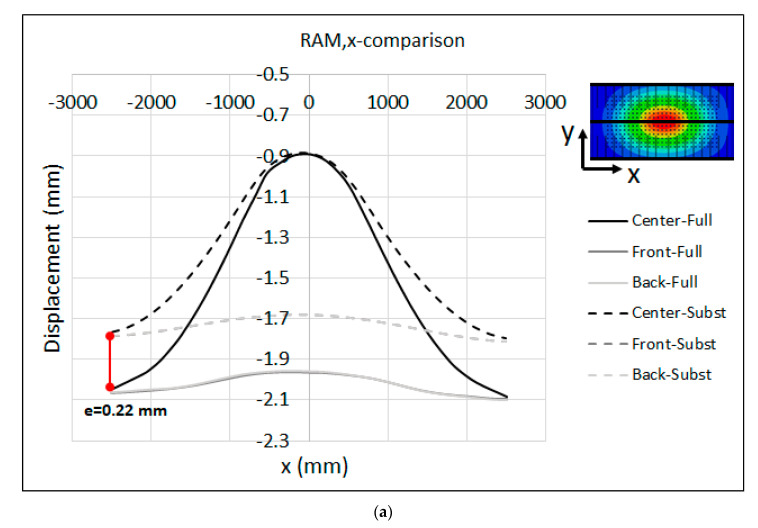

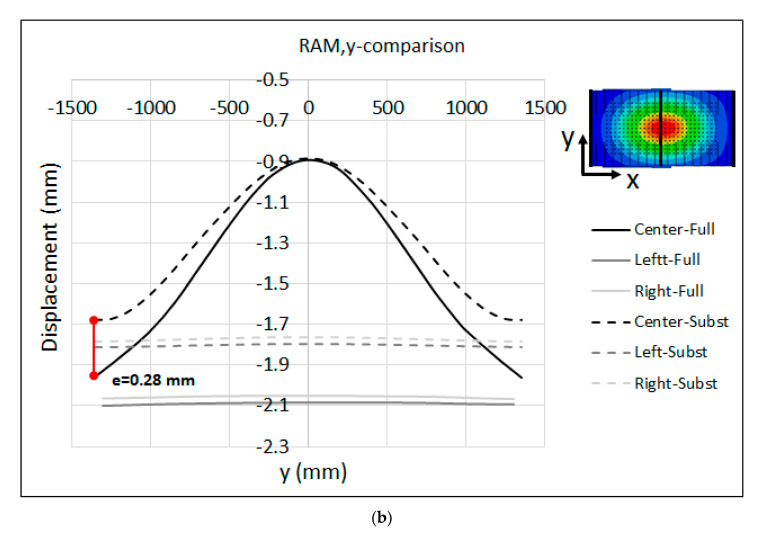
Substitute model vs. full-model displacement (adjusted) of the RAM along the (**a**) x-axis and (**b**) y-axis for the B-Pillar tool.

The displacement results of the ram along the x- and y-axes are shown in Figure 14. The maximum displacement at the center of the ram was about 0.91 mm. As seen in the figure, the substitute model estimated a lesser displacement for the ram with respect to the full model, indicating that the substitute model of the ram was stiffer than the full model. The maximum deviation was about e = 0.22 mm at the left edge of the centerline along the x-axis, and e = 0.28 mm at the left edge of the centerline along the y-axis. 

**Figure 15:**
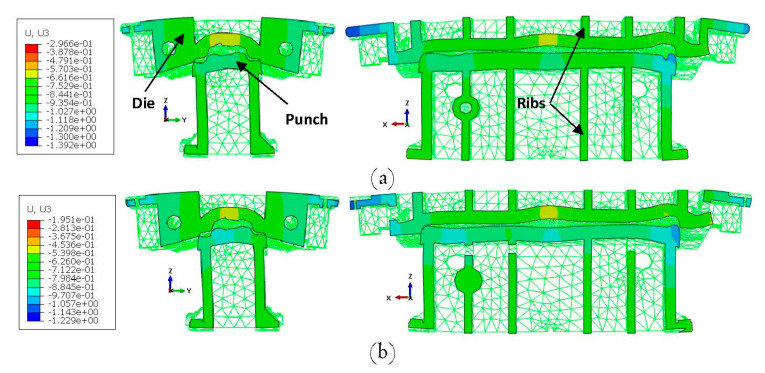
Deformation of the punch and die in the B-Pillar tool at the middle section along the x-axis (left images) and y-axis (right images) for (**a**) Full model and (**b**) Substitute model.

**Figure 16:**
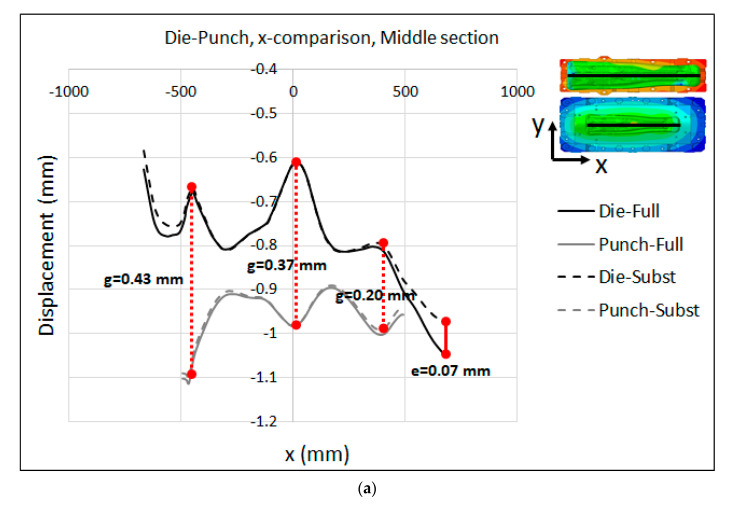

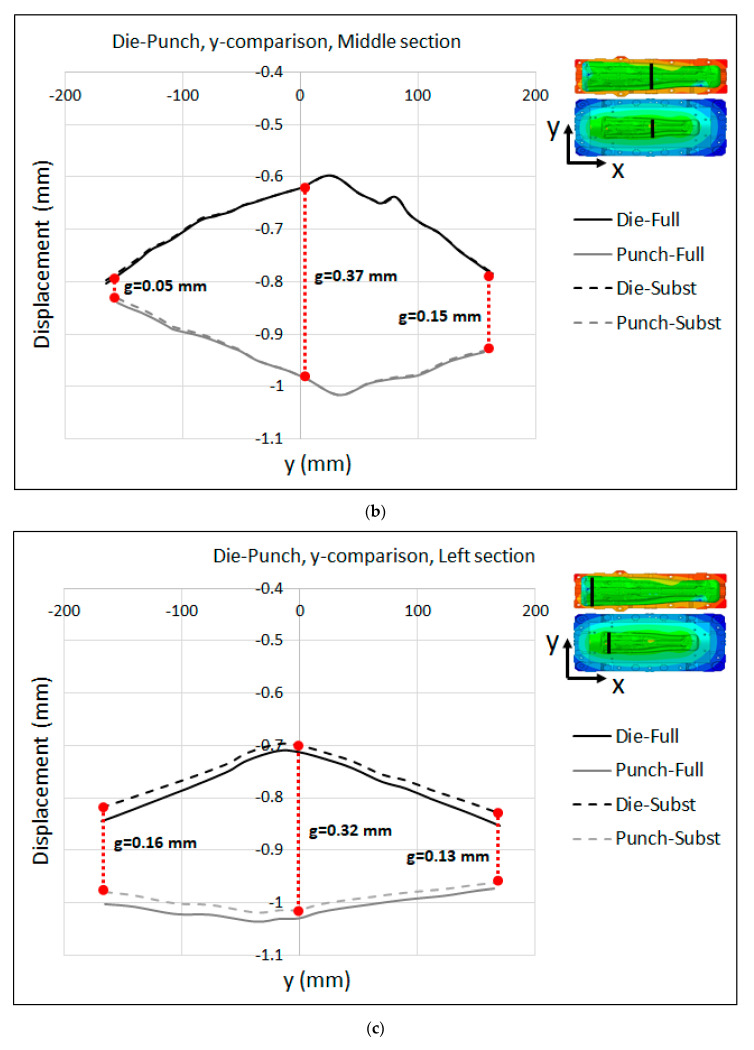

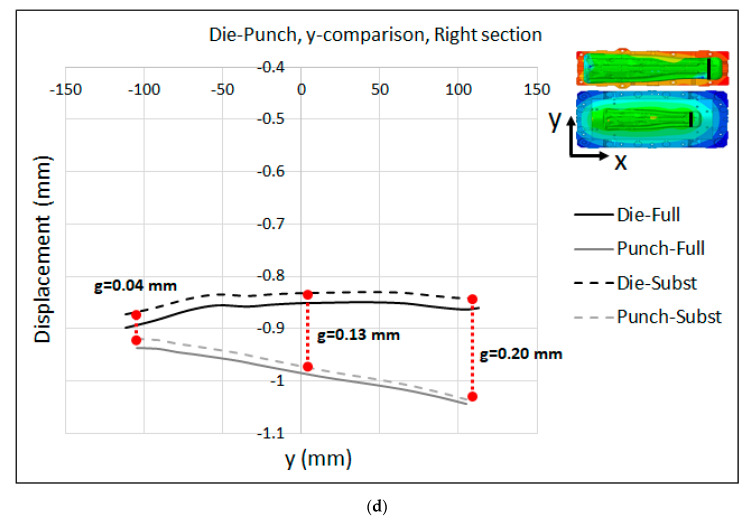
Substitute model vs. full-model displacement (adjusted) of the punch and die along different sections for the B-Pillar tool: (**a**) Middle section along x-axis (**b**) Middle section along y-axis (**c**) Left section along y-axis (**d**) Right section along y-axis.

The displacement distributions on the active surfaces of the punch and die at the middle section of the models along the x- and y-axes are presented in Figure 15a,b for the full model and substitute model, respectively. The displacement was magnified by a scale factor of 100 in order to further study the difference between the models and visualize the distance between the punch and die. As seen in the figures, the punch and die were deformed in opposite directions with respect to each other. This was clearer at the middle section along the y-axis (right images in Figure 15 where a large gap between the punch and die was produced at the center point. Moreover, by comparing the range of the displacement between the full model and substitute model, it can be concluded that there was a good agreement between the two models. For further validation of the substitute model, as well as the quantification of the gap between the punch and die, the comparison between the displacements in the active surfaces of the punch and die for both the full model and substitute model is illustrated in Figure 16 along the x- and y-axes, at different sections. Figure 16a shows a maximum deviation of e = 0.07 mm at the right edge and e = 0.04 mm at the front edge of the centerline. However, the displacements predicted by the substitute model and full-model simulation were almost identical for the punch. This suggests that the substitute model was able to precisely predict the deformation of the punch and die. A closer look at the results displayed in Figure 16 indicates a significance gap between the active surfaces of the punch and die that were directly related to the elastic behavior of the press and tooling. The elastic deformation along the contact interface between the punch and die amounted to a gap distance of g = 0.43 mm, g = 0.37 mm and g = 0.20 mm at X = −438 mm, X = 28 mm and X = 400 mm, respectively. The flat and valley pattern in Figure 16a is due to the complex geometry of the active surfaces of the punch and die. In addition, the peak values can be associated to the regions in the contact interface that are located between the ribs structure, where the stiffness is not enough to restrict the elastic deformations.

The comparison between the displacement in the active surfaces of the punch and die for both the full model and substitute model along the y-axis is illustrated Figure 16b–d for the middle, left and right sections, respectively. The results show a maximum deviation of e = 0.03 mm at the front edge along the left and right sections. However, the displacement predicted by the substitute-model and full-model simulations were almost identical along the middle section. The maximum elastic deformation along the contact interface between the punch and die amounted to a gap distance of g = 0.37 mm, g = 0.32 mm and g = 0.13 mm at the center point of the interface along the middle, left and right sections, respectively. The asymmetrical clearance in displacement was due to the application of the rib structure to the die and punch.

**Figure 17:**
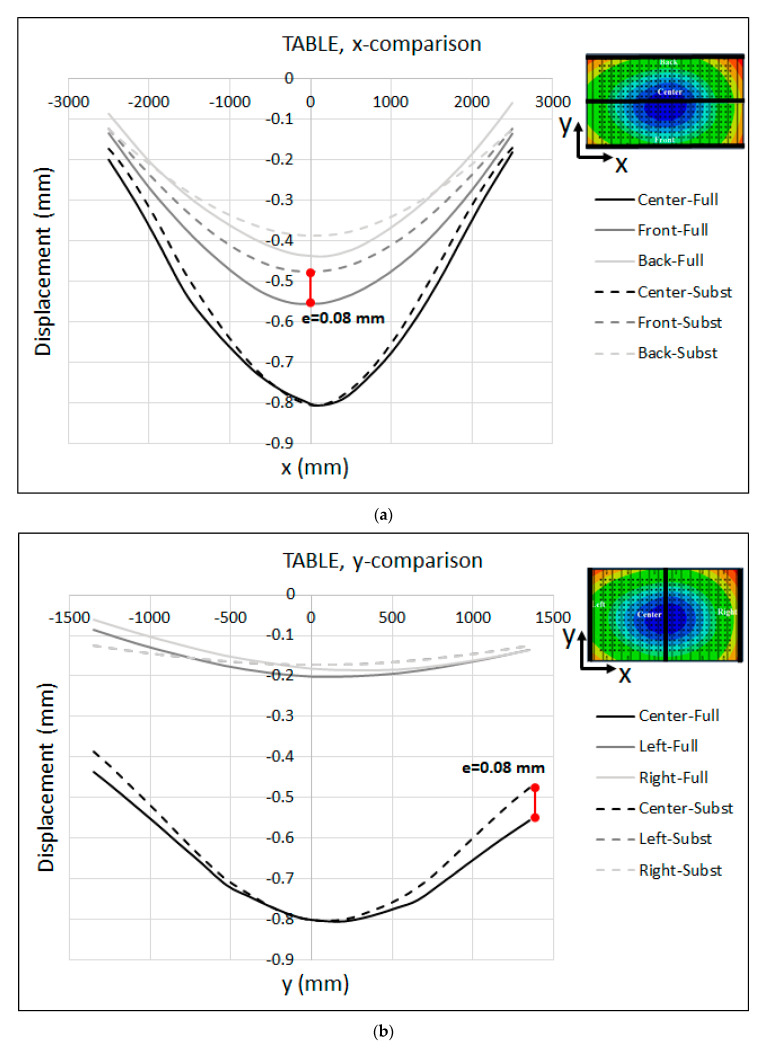
Substitute model vs. full-model displacement (adjusted) of the press TABLE along the (**a**) x-axis and (**b**) y-axis for the Lateral tool.

In order to validate the robustness of the substitute model against large-size tools (that are more sensitive to cambering), a side panel, called lateral (see Figure 4b), was modeled together with the substitute model, and the deformations of the press and tool were compared with the full-model-simulation results. Just as with the B-pillar tools, the substitute model of the press with the calibrated parameters that were previously determined in Section 4 was used. The comparison between the displacements predicted by the substitute-model and full-model simulation for the press table in the x- and y-axes is illustrated in Figure 17.

**Figure 18:**
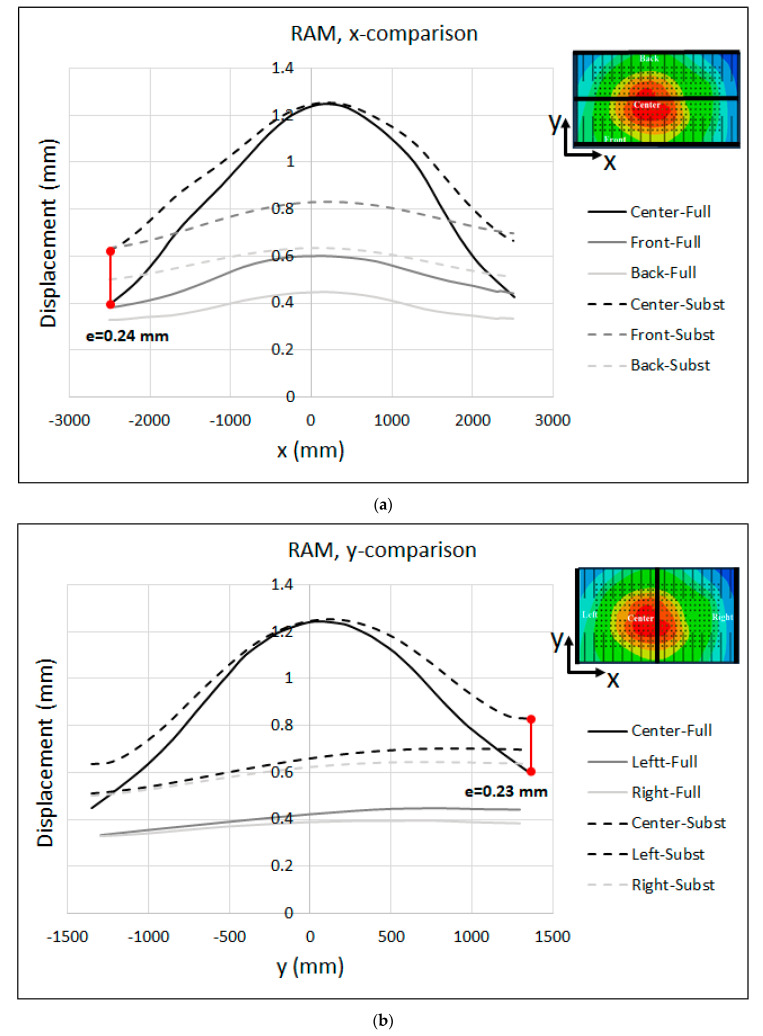
Substitute model vs. full-model displacement (adjusted) of the RAM along the (**a**) x-axis and (**b**) y-axis for the Lateral tool.

The maximum displacement at the center of the table was about 0.8 mm. As seen in the figure, the displacements predicted by the substitute model were in quite good agreement with the full-model-simulation results, especially in x-direction, where both models predicted the same shape along the centerline. However, the maximum deviation of e = 0.08 mm appeared at the middle of the front edge along the x-axis and the right edge of the centerline along the y-axis. Figure 18 shows the same results for the ram. The maximum displacement at the center of the table was about 1.25 mm. Furthermore, the maximum deviation between the substitute-model and full-model simulations was about e = 0.24 mm at the left edge of the centerline along the x-axis, and e = 0.23 mm at the right edge of the centerline along the y-axis. The deviation between the substitute-ram-model and the full-model-simulation results shown in Figure 14 and Figure 18 can be attributed to the shell thickness in the substitute model, which corresponded to the real dimensions of the press table and ram. In the full-model simulation, both the table and mounting surfaces of the ram contained holes and sleeves that were neglected in the substitute models. In this context, an optimization approach has to be developed in order to find the effective thickness of the shell elements in the substitute model.

**Figure 19:**
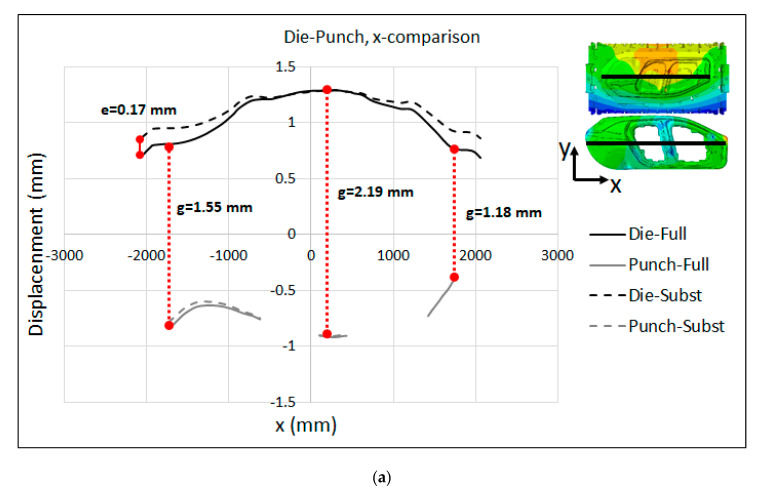

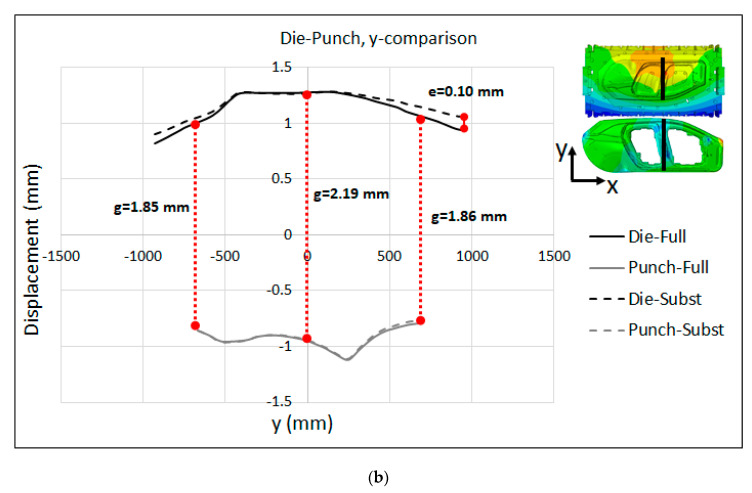
Substitute model vs. full-model displacement (adjusted) of the punch and die along the (**a**) x-axis and (**b**) y-axis for the Lateral tool.

Relative to the displacement distributions on the active surfaces of the tooling, the comparison between the displacements on the active surfaces of the punch and die for both the full model and substitute model are illustrated in Figure 19a,b along the x- and y-axes, respectively. It can clearly be seen in the figure that the punch and die are deformed in opposite directions with respect to each other, providing evidence for the significance of the effects of press and tool elasticity on the industrial sheet-metal-forming process and real production tools. There was a very good agreement between the results predicted by both models, especially for the punch, for which the displacements predicted by the substitute-model and full-model simulations were almost identical along both the x- and y-axes. This is because the displacement of the punch was mainly dominated by the elastic deformation of the press table, for which the substitute model has less deviation with respect to the ram, as shown in Figure 17 and Figure 18.

Regarding the displacement on the active surface of the die, Figure 19a,b show a maximum deviation of e = 0.17 mm at the left edge and e = 0.10 mm at the back edge of the centerline. Moreover, the maximum and minimum gap distances between the punch and die along the contact interface amounted to g = 2.19 mm and g = 1.18 mm at the middle and right edge of the centerline, respectively. In a real production, these gaps are the main cause of non-homogeneous pressure distribution on the equipment and must be compensated by manual rework during the trial in order to achieve a precise tool closure. Within the scope of the present study, accurate prediction of the final gap between the punch and die is a crucial factor for the employment of the new cambering strategy that is proposed in Section 6.2.

In summary, it is worth mentioning that the computational time is enormously increased when including the elastic behavior of the press and tools, especially for complicated stamping parts in the early design stages before the trial phase. In this context, the new substitute model proposed in this study aims to remarkably shorten the calculation time while keeping the accuracy of the results. To shed light on the issue, the CPU conditions and time consumed for each simulation are listed in Table 2 for both the full model and the substitute model developed in this study. As seen in Table 2, the computational time of the FE substitute press model was reduced by approximately 96%, 77% and 72% for the four-pillars, B-pillar and Lateral tools, respectively.

### 6.2. Real Cambering of the B-Pillar Tool and Reduction of Trial Time

The B-Pillar tool that was available at Matrici S. Coop (the European leading company in dies and stamping tools for skin parts) was re-engineered with respect to the numerical results presented in the previous section. In order to quantify the influence of the cambering on the final product accuracy and to estimate the possible reduction in the trial time, two types of stamping were performed.

**Figure 20:**
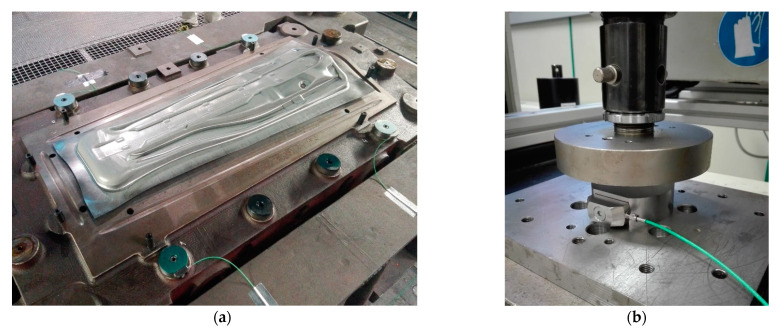
(**a**) Stop blocks equipped with strain sensors (blue painted) and (**b**) calibration procedure of the stop block using a universal compression machine.

First, both the punch and die were machined using the nominal geometry and by applying a gap equal to the sheet thickness between them. Furthermore, all of the tools, including the punch, die and binder, were manually polished to the standard level used in the Matrici S. Coop. During the first stampings, a novel methodology was used to adjust the stop blocks that control the closing between the binder and die. Piezoelectric strain sensors from Kistler were used in four corners of the die in order to monitor the stop-block forces. The stop blocks were gauged until an acceptable balance of the closing force was found in the tool. The location of the sensors and the calibration of the stop blocks, which were performed in a universal compression machine, are shown in Figure 20a,b.

**Figure 21:**
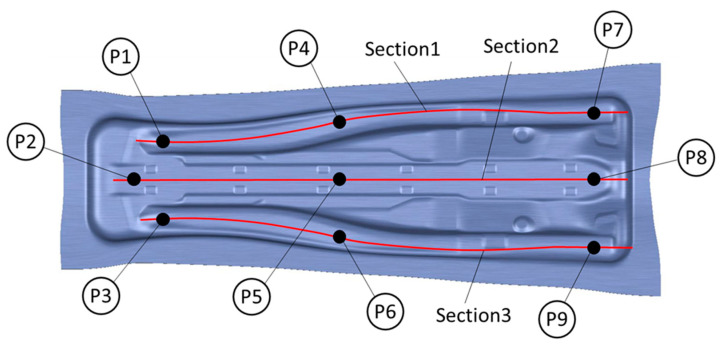
Lead check measuring points and sections used for die morphing.

Once the stop blocks were balanced, the stampings were performed using Fortiform 1050 third generation steel precuts. In order to measure the cambering effects, a lead check was performed on nine points of the tool (see Figure 21). The use of lead pellets enables the measurement of the real gap between the die and punch at the closed state of the tool, when the precut is also included and suffers from thinning.

Secondly, the tool die was digitally cambered using the morphing option called Wrap Curve of the CATIA software. The cambering strategy was decided by the Matrici S. Coop after the numerical results of the substitutive models and the thinning values of AutoForm were analyzed. The punch geometry was not modified, and the sum of both elastic deflections suffered by the die and punch were applied to the die morphing.

**Figure 22:**
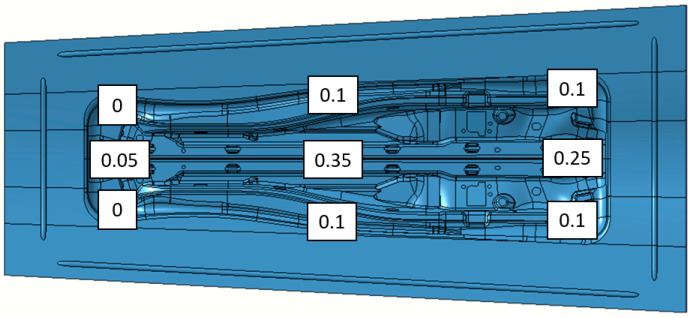
Control points that define the three splines for the cambering of the die using the Wrap Curve operation from CATIA.

The Wrap Curve operation of CATIA employs master curves to morph the surfaces. Three splines were defined (one in the top, one in the middle and one in the bottom of the die) for this purpose (see Figure 21). The vertical displacement of the control points of the three splines are detailed in Figure 22. Note that the cambering is not symmetrical within the vertical axis, which is the usual procedure used by the Matrici S. Coop for these types of reinforcement components.

The same procedure as the one used with the nominal gap tooling was used in the second stamping trial. The lead check was performed on the same points with the new cambering methodology after the stop blocks were balanced using the force measurements. All of the lead check results are shown in Table 3.

The real gap measurements suggest that the new cambering methodology based on numerical results obtained from the substitutive models was able to reduce the trial time of the tooling. Even if the tool is a B-Pillar and small in comparison to other tools that are more sensitive to cambering, it was confirmed that at least one re-machining and polishing loop could be avoided with the new strategy. This can be understood by the analysis of the errors observed in each of the sections used in the virtual cambering.

Section one, which is defined by the points P1, P4 and P7, displays a maximum deviation in the vertical z-axis of 0.1 mm in the nominal gap tests in contrast with the error of 0.03 mm that was observed for the new cambering method. The same results were obtained for the third section, which is defined by the points P3, P6 and P9 of the tool. It is worth mentioning that without considering the central section, all of the tools would be within a gap error of 0.03 mm. It is likely that no polishing would be needed in order to obtain a good blue-spotting pattern.

The central section, section two, which is defined by the points P2, P5 and P8, exhibits a maximum error of 0.3 mm for the nominal gap tooling and 0.16 mm of error for the new cambering method. Although a significant improvement was achieved, the new cambering method was not able to fully correct the error due to the elastic deformation and thinning of the sheet. One new optimization loop by cambering the central point with 0.16 mm would probably yield a perfect contact between the punch, sheet and die, thereby offering a good blue-spotting pattern. On the contrary, having error in the two directions of the tool, such as in the nominal gap trials, would probably have required two new optimization loops and tool re-engineering.

## 7. Conclusions and Future Lines

A simple and cost-effective press-substitute model composed of low-cost shell and beam elements was developed for a 20,000 kN trial press. The newly developed substitute model was calibrated using full-scale measurements in which the trial press was experimentally characterized by measuring its elastic deformation under static loads. To evaluate the robustness of the substitute model, a medium-size tool (B-pillar) and a large-size (Lateral) tool were simulated together with the substitutive press model and the deformations in the table, ram and active surfaces of both tools were compared with the full-model-simulation results. The results showed the significance of the effect of the press elasticity on the industrial SMF process and real production tools. Moreover, the substitute model was able to replicate the stiffness of the press regardless of the size of the tooling. The global shapes of the deformed table and ram were also captured satisfactorily by the proposed substitute model. The maximum displacements at the center of the press table were found to be 0.71 mm and 0.8 mm for the B-pillar and Lateral tools, respectively. At the middle of the ram, the maximum displacement amounted to 0.91 mm and 1.25 mm for the B-pillar and Lateral tools, respectively. Moreover, the maximum clearance gaps between the active surfaces of the punch and die were 0.43 mm and 2.19 mm for the B-pillar and Lateral tools, respectively. By implementing the displacement obtained from the substitute model together with the thinning values obtained from AutoForm, new cambering strategies were developed in which the B-pillar reinforcement die was re-engineered at Matrici S. Coop.

The main advantages of the newly developed substitutive model and the new model-calibration methodology are that the real 3D geometry of the press is not necessary, and the calibration procedure is very straightforward. In this way, the press-specific substitute model proposed in this study can be generated for any type of measured press. In addition, the computational time associated with the FE-substitute-press model was reduced by approximately 96%, 77% and 72% for the four-pillars, B-pillar and Lateral tools, respectively. This is an additional important advantage of the proposed substitute model that can aid toolmakers in shortening the virtual design and eliminate most of the reworking and home-line efforts at the early stages of the trial. In order to quantify the influence of cambering on the final product accuracy and estimate the possible reduction of the trial time, two types of stamping were performed on a B-Pillar reinforcement tool using the numerical results obtained from the substitute model. It was found that the new cambering methodologies based on the numerical results obtained from the substitutive models were able to significantly reduce the trial time of a tooling.

As for future lines of work, it is suggested to measure the displacements of all four supports of the measuring frame relative to the bolster in order to both improve the accuracy of the measuring system and to obtain the absolute deformation along the press table and ram. Additionally, by using the deflection of the bolster and ram as a target, it is preferable to calibrate the stiffness of the substitute model (stiffness of the beam elements and effective thickness of the shell elements) during the iterative parameter optimization of the model in ABAQUS and MATLAB in order to both enhance the accuracy of the substitute model and to reduce the deviation between the full-model and substitute-model results. The guiding columns of the press were not included in the FE models for the sake of simplicity, hence further studies are needed to investigate the effect of press columns on global stiffness and the deformation of the entire press bolster and ram. Finally, a meshless approach [17,18] can be implemented on the complex contacts of the active surfaces of tools where mesh distortion provokes a significant loss of accuracy in the results. 

## Figures and Tables

**Table 1:** Material parameters used in FE simulations.

Material	Density (kg/m^3^)	Young’s Modulus (GPa)	Poisson’s Ratio (-)
Steel	7800	210	0.3
GG70L	7200	175	0.33

**Table 2:** Overview of the computational time consumed for each model.

Tool Type	CPU Model	Number of Parallel CPU	Computational Time		Redaction Percentage (%)
4-pillars	Intel Core i9-10980XE CPU@3.00 GHz	8	Full model	Substitute model	96
			60 min	2 min	
B-pillar	Intel Core i9-10980XE CPU@3.00 GHz	8	Full model	Substitute model	77
			13 h	3 h	
Lateral	Intel Core i9-10980XE CPU@3.00 GHz	8	Full model	Substitute model	72
			22 h	6 h	

**Table 3:** Lead-check test measurements.

Point Number	Real Gap without Cambering (mm)	Real Gap with New Cambering Strategy (mm)	Section Number	Maximum Error in Horizontal Sections without Cambering (mm)	Maximum Error in Horizontal Sections with New Cambering (mm)
P1	0.18	0.18	Section 1	0.1	0.03
P2	0.18	0.18			
P3	0.18	0.18			
P4	0.28	0.18	Section 2	0.3	0.16
P5	0.48	0.3			
P6	0.28	0.18			
P7	0.19	0.15	Section 3	0.1	0.03
P8	0.19	0.14			
P9	0.19	0.21			

## Data Availability

No extra data produced.
